# Effects of variable mating opportunity, delay, and male mating experience on the lifespan, female reproductive traits, and offspring traits of *Phytoseiulus persimilis* (Acari: Phytoseiidae)

**DOI:** 10.1007/s10493-025-00999-8

**Published:** 2025-01-28

**Authors:** Xia Chen, Keshi Zhang, Zhi-Qiang Zhang

**Affiliations:** 1 Institute of Plant Protection, Fujian Academy of Agricultural Sciences, Fuzhou, Fujian China; 2 Manaaki Whenua – Landcare Research, 231 Morrin Road, Auckland, 1072 New Zealand; 3 School of Biological Sciences, University of Auckland, Auckland, 1072 New Zealand

**Keywords:** Biocontrol, Copulation frequency, Predator, Reproduction

## Abstract

The balance between mating benefits and costs shapes reproductive strategies and life history traits across animal species. For biological control programs, understanding how mating rates influence life history traits is essential for optimising population management and enhancing predator efficacy. This study investigates the impact of mating opportunity availability, delayed mating, and male mating history (copulation frequency) on the lifespan (both sexes), female reproductive traits (duration of oviposition and of pre- and post-oviposition periods, and lifetime oviposition), and offspring quality (egg size and offspring survival) of the predatory mite *Phytoseiulus persimilis* Athias-Henriot (Acari: Phytoseiidae), an important biological control agent against spider mites. We examined three mating treatments—no mating, limited mating opportunity (24-h access), and continuous lifetime access—to assess their effects on lifespan (both sexes), female reproductive traits, and offspring quality. Further analyses examined the impact of delayed mating and male copulation history on female reproductive success and offspring traits. Our results showed a sexually differentiated response to repeated mating: females with continuous access to mates had similar lifespans in comparison with those mated for only 24 h, while males with continuous mating access exhibited significantly shorter lifespans. Both unlimited mating and delayed mating prolonged the female pre-oviposition period. However, neither varied mating opportunities, delayed mating, nor male copulation had any significant effect on other female reproductive traits or offspring quality. This suggests that repeated mating provides no reproductive advantage and imposes no observable costs on *P. persimilis* females.

## Introduction

Optimal mating rates often vary between females and males in sexually reproducing species (Arnqvist and Nilsson 2000). For males, reproductive success would typically increase with more mates, as mating with multiple females could enhance their potential for offspring production (Arnqvist and Nilsson 2000; Yokoi et al. 2023). In contrast, females maximize their reproductive output by increasing egg production (Arnqvist and Nilsson 2000). Mating, however, involves considerable energetic and time costs, and it can increase exposure to predation, injury, and the risk of parasite or disease transmission (Barbosa et al. 2012; Chapman et al. 1998; Djawdan et al. 1996; Härdling and Kaitala 2005). Therefore, females are generally expected to require minimal mating events to achieve optimal reproductive success (Arnqvist and Nilsson 2000).

Despite this expectation, polyandry (mating with multiple males) and repeated mating with the same male are behaviors observed more frequently than expected in many animal species (Arnqvist and Nilsson 2000). Multiple mating offers direct benefits to females, such as increased fecundity due to fresh sperm supplies, addressing insufficient sperm from previous matings, and the physiological stimulation associated with mating itself (Barbosa et al. 2012; Härdling and Kaitala 2005). Furthermore, multiple matings can enhance offspring fitness by increasing genetic diversity, facilitating sperm competition, and enabling post-copulatory sexual selection, which can lead to more competitive offspring (Barbosa et al. 2012; Collet et al. 2012; Härdling and Kaitala 2005; Radwan 1997). Despite a direct positive influence on fecundity, increased mating events often result in a reduced lifespan for females in a diverse range of animal species, possibly due to the costs of reproduction and the physiological impacts of mating itself (Arnqvist and Nilsson 2000; Chapman et al. 1998; Dunn et al. 2005; Nakagawa et al. 2012; Ridley 1988).

The family Phytoseiidae (Acari: Mesostigmata) contains important plant-inhabiting biological control agents that are widely used in agriculture to manage mite and insect pests (McMurtry et al. 2013, 2015; Zhang 2003). Understanding factors influencing reproduction and oviposition in phytoseiids has attracted significant interest due to their critical role in successful biological control programs (Amano and Chant 1978; Su et al. 2023). In most phytoseiid species, both mating and insemination by males are required to initiate female reproduction (Ji et al. 2007; Norton et al. 1993; Pappas et al. 2007; Saber and Momen 2000; Toyoshima et al. 2000). Furthermore, multiple and repeated matings are frequently observed within this family (Amano and Chant 1978; Momen 1997; Schulten et al. 1978; Tsunoda and Amano 2001).One of the few exceptions is *Phytoseiulus persimilis* Athias-Henriot (Acari: Phytoseiidae), a specialist predatory mite utilised for decades to control spider mites, particularly the two-spotted spider mite (*Tetranychus urticae* Koch) (Trombidiformes: Tetranychidae) and its close relatives (Bajda et al. 2022; McMurtry et al. 2013). In *P. persimilis* females, mating frequency is relatively low, with a single mating event being sufficient to reach maximum reproductive output (Amano and Chant 1978; Schausberger et al. 2017). Additional matings do not increase fecundity or extend the oviposition period, but they do reduce the lifespan, particularly the post-oviposition phase, of *P. persimilis* females (Amano and Chant 1978).

Apart from mating frequency, delayed mating and male fertility also could influence the reproductive success and lifespan of females (Li and Zhang 2021a; Yokoi et al. 2023). Delayed mating after sexual maturity has been observed to extend lifespan and reduce daily oviposition rate in some species, as seen in *T. urticae* females when mating was postponed for seven days (Li and Zhang 2021a). In contrast, in other species, such as the phytoseiid predator mite *Neoseiulus californicus* even a delay of up to 42 days showed no significant impact on its lifespan, fecundity, or the oviposition period (Gotoh and Tsuchiya 2008). The capacity of males to mate with multiple females depends on the availability and replenishment rate of sperm (Arnqvist and Nilsson 2000; Yokoi et al. 2023). For example, *T. urticae* males could replenish their sperm supply within 3 h (Yokoi et al. 2023). However, it has been suggested that the reproductive capacity of *P. persimilis* males declines after the first mating (Tsunoda and Amano 2001). Therefore, this study investigated the influence of delayed mating (or age at mating) and male mating history on male and female lifespan, female reproductive traits (including pre-oviposition period [duration of adulthood before oviposition], oviposition period [first to the last day of oviposition], post-oviposition duration [last ovipositional day to death], and fecundity [lifetime oviposition] of females), as well as offspring survival in *P. persimilis*.

Most researches on Phytoseiidae have been focused on the females, particularly lifespan and egg quantity, with less emphasis on the effects of multiple mating on males (e.g., Ji et al. 2007). This study has aimed to address this gap by investigating the impact of repeated mating (with the same partner) on the lifespan of *P. persimilis* males. Additionally, the study examines the influence of repeated mating on offspring quality, including egg size and survival rates to adulthood. Given that multiple inseminations are unnecessary for maximal reproduction in *P. persimilis* females, repeated mating by males might act as harassment, negatively impacting females (Härdling and Kaitala 2005; Li and Zhang 2021b). We hypothesized:

1. The influence of repeated mating on lifespan would differ between the sexes, with more negative effects for females than males.
2. Due to the supplement of fresh sperm supply, repeated mating would positively affect offspring traits, leading to bigger egg size and increased survival rates to adulthood.
3. Delayed mating would have little influence on the lifespan and reproductive output of *P. persimilis* females, and this would be similar to the patterns observed in *N. californicus*.
4. Females inseminated by previously mated males would show reduced reproductive success, lower offspring quality, and altered offspring sex ratios.

The results of this study would provide insights that enhance our understanding of the life history and reproductive strategies of *P. persimilis*, potentially informing more effective biological control strategies for spider mites in agricultural settings.

## Materials and methods

### Mite cultures

The predator species, *P. persimilis*, was reared under controlled laboratory conditions at Manaaki Whenua—Landcare Research, Auckland, New Zealand. The laboratory maintained a consistent temperature of 20 ± 2 °C, with relative humidity (RH) between 65 and 75%, and a 12-h light/dark photoperiod (see Han et al. 2022 for further details). The prey species, *T. urticae*, was reared on bean plants (*Phaseolus vulgaris* L.) with the same laboratory conditions as the predator. Bean leaves infested with *T. urticae* were regularly harvested and supplied as prey for *P. persimilis*. Initial populations of both *P. persimilis* and *T. urticae* were obtained from Bioforce Limited (Karaka, Auckland, New Zealand).

For the experiment, numerous gravid *P. persimilis* females were taken from the culture and placed on fresh *T. urticae*-infested bean leaves to oviposit. Newly laid eggs (< 24 h old) were collected and used the following day for the experiments. Similarly, gravid *T. urticae* females were transferred to fresh bean leaves for oviposition, and newly laid *T. urticae* eggs (< 24 h old) were supplied as prey for *P. persimilis* throughout the experiment.

### Rearing units

We used rearing cells for immature mites and for pairing/mating of males and females the same as in Han et al. (2022). Each cell comprised a plexiglass slide with a central 6 mm cavity, covered by a glass slide to accommodate the test subjects. A black cotton cloth was stuck to the base of the plexiglass slide to provide contrast for mite observation and ventilation. Metal clips secured the slides together.

For adult rearing, 0.5 mL centrifuge tubes were modified by adding a central hole in the lid, covered with black cloth for ventilation (Xu and Zhang 2015; Xu et al. 2024). Bean leaf squares infested with *T. urticae* eggs were placed on T-shaped filter paper inside the tubes. Filtered water (c. 10 μL) was added to the filter papers daily to maintain leaf freshness. The rearing tubes were stored in sealed containers with saturated salt water to maintain approximately 80% RH, and the setup was kept in an incubator at 25 ± 2 °C (Lee et al. 2020).

### Experiment 1a: Influence of mating opportunity on lifespan, female reproductive traits, and offspring survival

**Fig. 1:**
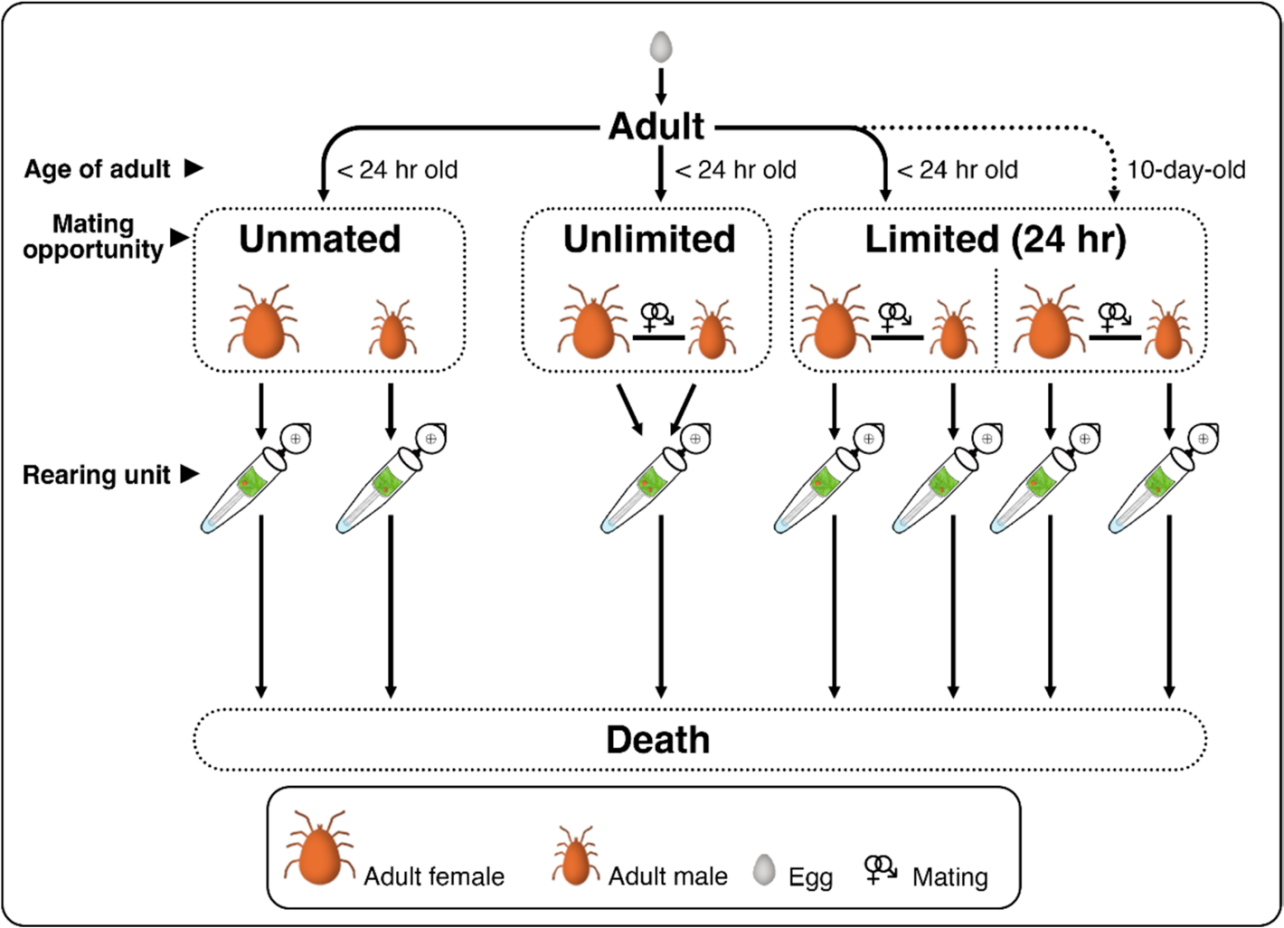
Schematic of the experimental setup examining the influence of mating opportunity and delayed mating on the lifespan and reproduction of *Phytoseiulus persimilis* in Experiments 1a and 1b. The dotted line for 10-day olds refers to Experiment 1b

Newly laid *P. persimilis* eggs (< 24 h old) were individually reared in rearing cells. Females of *P. persimilis* were about 20% larger than males (Han et al. 2022). Upon reaching adulthood, individuals were sexed, and adults (< 24 h old) were randomly assigned to one of three mating treatments. Each treatment had 15–18 replicates per sex. The treatments (1–3) are described below and shown in Fig. 1:

1. Unmated: Adults were transferred to individual rearing tubes and kept without mating.
2. Unlimited mating opportunity: Pairs of adults were allowed to mate for 24 h in rearing cells before being transferred to the same rearing tube to permit continuous mating until death. If one individual died of natural causes unrelated to experimental manipulation (e.g., not drowned in water or lost during feeding), the surviving individual remained in the same rearing tube and continued to be observed until its natural death.
3. Limited mating opportunity: Pairs mated for 24 h in rearing cells were separated and transferred to individual rearing tubes to examine the effects of limited mating exposure on longevity and reproductive traits.

Throughout immature development and mating, an ad libitum supply of *T. urticae* eggs was provided as prey. For adult *P. persimilis*, bean leaf squares infested with *T. urticae* eggs were replenished every two days or as needed to provide an ad libitum supply of prey.

Daily checks recorded lifespan of both males and females, as well as pre-oviposition period, oviposition period, post-oviposition duration, and fecundity of females. Egg size (> 30 per treatment) was measured using interference-phase contrast microscopy (SMZ25, Nikon Corporation, Japan) at 200× magnification. The size of the eggs was measured as individual egg volume following the formula provided by Narushin (2005). The individual egg volume (*V*) was calculated using the length (*L*) and maximum breadth (*B*) of the eggs:

$$ {\text{V  =  (0}}{\text{.6057}} - {\text{0}}{\text{.0018B)LB}}^{2}  $$

After measuring their size, 30 eggs from each treatment were individually reared to adulthood, as previously described, to assess offspring survival (proportion of individuals that survived to adulthood) and sex ratio (proportion of females).

### Experiment 1b: Influence of delayed mating on lifespan, female reproductive traits, and offspring survival

Fifteen recently moulted (< 24 h old) females and males of *P. persimilis* were continuously reared individually for 10 days before being paired for mating for 24 h (Fig. 1). After mating, the adults were separated and transferred to individual rearing tubes. The same traits as in Experiment 1a were measured.

### Experiment 2: Influence of male copulation history on lifespan, female reproductive traits, and offspring survival

**Fig. 2:**
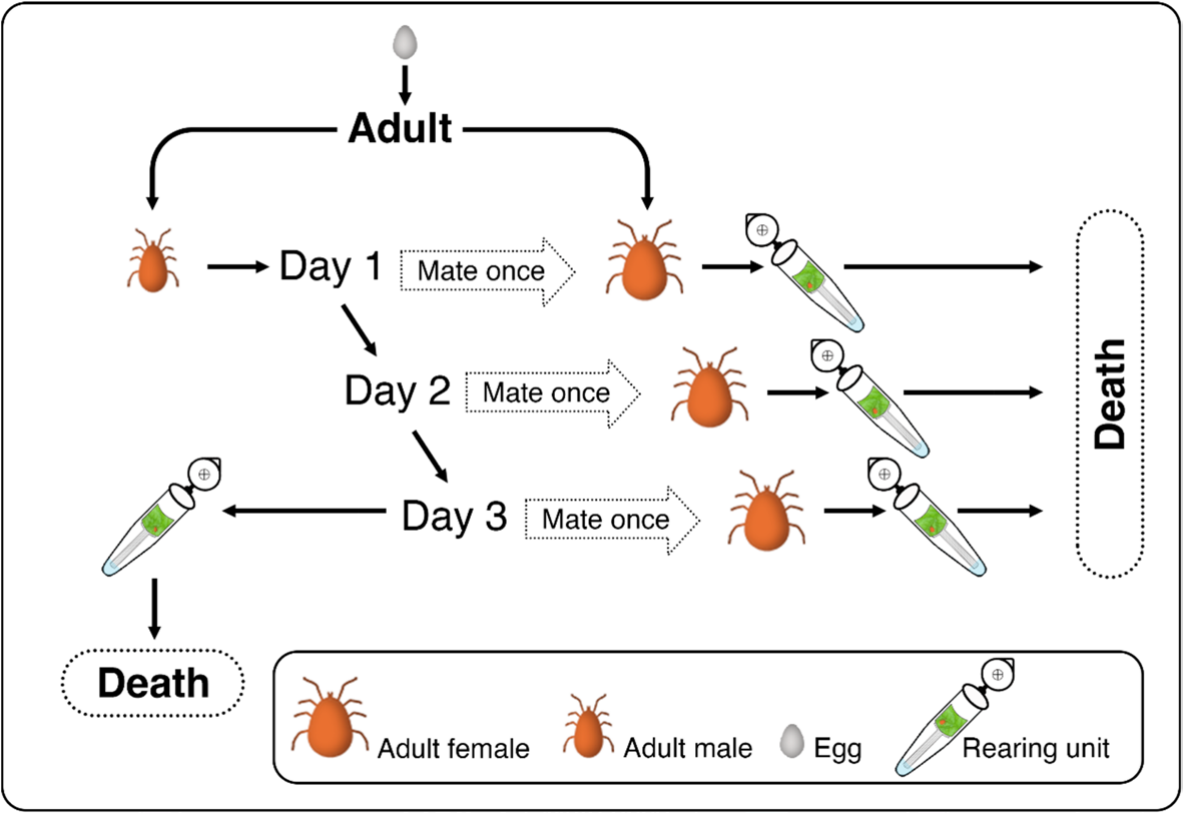
Schematic of the experimental setup examining the influence of male copulation history on the lifespan and reproduction of *Phytoseiulus persimilis*

Twelve newly moulted *P. persimilis* males (< 24 h old) were each allowed to mate with 3 different females (< 24 h old) across 3 consecutive days (Fig. 2). Following each mating event, males were returned to their rearing cells, and females were transferred to individual rearing tubes. After three mating events, males were individually reared in rearing tubes. Lifespan and reproductive traits were measured as described in Experiment 1a.

### Statistical analysis

All statistical analyses were conducted in R (R Core Team 2022) using R studio (version 2023.12.1). Data visualisation was performed using the *ggplot2* package (version 3.4.3) (Wickham 2016), and statistical significance was determined using the *ARTool* package (version 0.11.1) (Kay et al. 2021). Four individuals in Experiment 1 and five in Experiment 2 that were lost during the experiment, due to experimental errors, were excluded from further analysis. Data were summarised using means and standard errors (SEMs). Egg sizes were presented using box plots that display the interquartile range (IQR; middle 50%), the median, and the data spread (1.5 times the IQR). Outliers, if any, were retained unless noted otherwise. SEMs of offspring survival rate and sex ratio were caluculated by the fomula: ${\text{SEM  =  v(P(1}} - {\text{P)}} \div {\text{n)}}$, using means (*P*) and sample sizes (*n*). Due to non-normal distributions, aligned ranks transformation (ART) analyses of variance (ANOVAs) and Wilcoxon rank-sum tests were applied to compare lifespan, pre-oviposition, oviposition, post-oviposition, fecundity, and egg size. Chi-square tests were used to assess survival rate and sex ratio in offspring. Statistical significance was set at *P* < 0.05.

## Results

### Experiment 1a: Influence of mating opportunity on lifespan

**Fig. 3:**
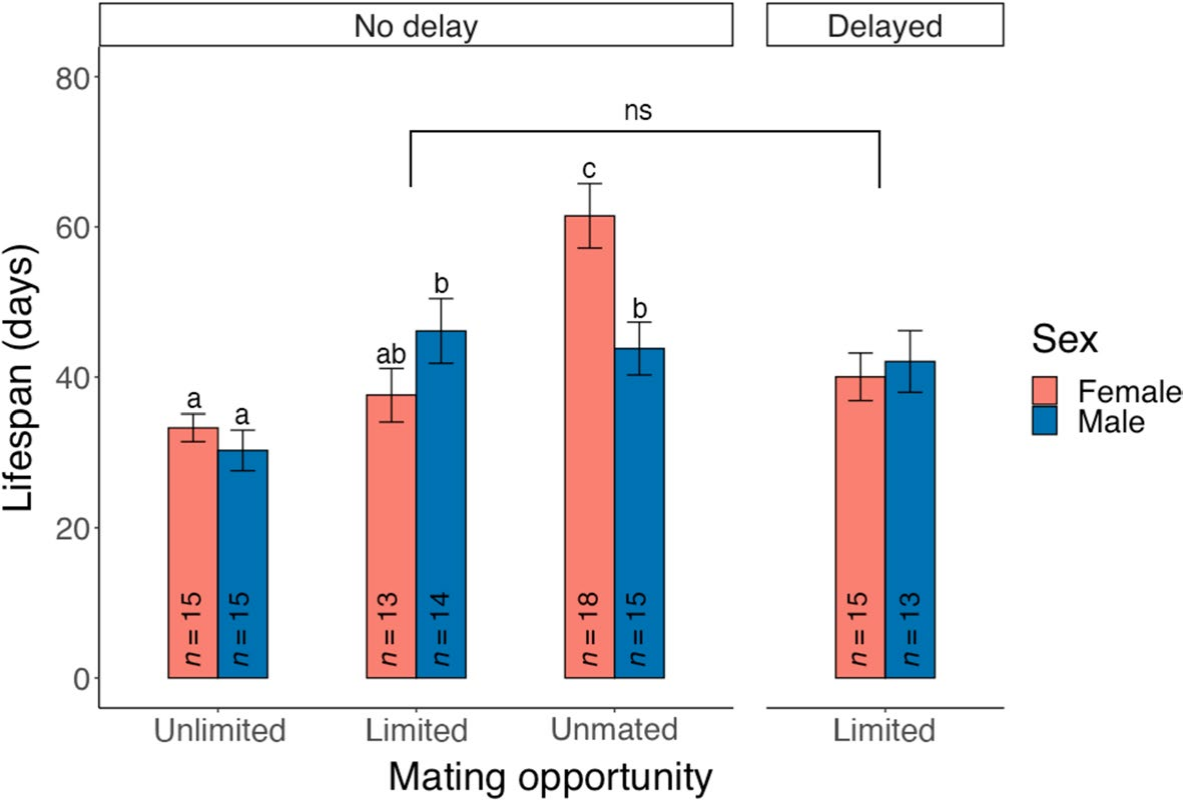
Influence of mating opportunity and delayed mating on the lifespan of *Phytoseiulus persimilis*. Individuals given limited mating opportunities were allowed to mate for 24 h. The age of adults at mating was either < 24 h (no delay) or 10-day-old (delayed). Different letters indicate significant differences between treatments (ART ANOVA pairwise comparisons: *P* < 0.05). For individuals given limited mating opportunities, delayed mating had no significant (ns) influence on the lifespan (*P* > 0.05). The bars error bars indicate means and SEM

Mating opportunity significantly affected the lifespan of *P. persimilis* (ART ANOVA: *F*_2,84_ = 19.685, *P* < 0.001). Individuals with multiple mating opportunities had the shortest lifespan (Fig. 3). Females generally lived longer than males (*F*_1,84_ = 6.140, *P* = 0.015), with the largest difference observed in the unmated group (Fig. 3). There was a significant interaction between sex and mating treatment (*F*_2,84_ = 6.900, *P* = 0.002). Specifically, unmated females had the longest lifespan, whereas males given unlimited mating exhibited a significantly reduced lifespan compared to those mated for 24 h or those not mated (Fig. 3).

### Experiment 1b: Influence of delayed mating on lifespan

The lifespan of individuals given limited mating opportunities was not significantly affected by the delayed mating (ART ANOVA: *F*_1,51_ = 0.106,* P* = 0.746) and sex (*F*_1,51_ = 6.140,* P* = 0.015), with no significant interaction between the two factors (*F*_1,51_ = 0.356,* P* = 0.533) (Fig. 3).

### Experiment 1a: Influence of mating opportunity on female reproductive traits and offspring survival

**Fig. 4:**
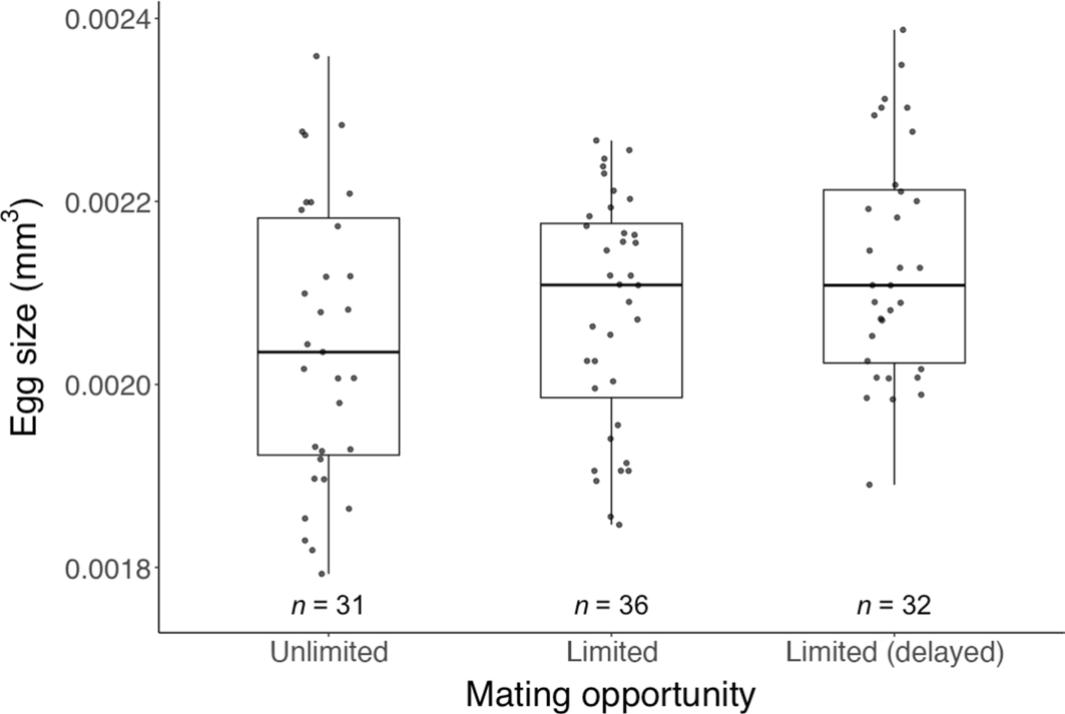
Influence of mating opportunity and mating delay on the egg size (measured as individual egg volume) of *Phytoseiulus persimilis*. Individuals given limited mating opportunities were allowed to mate for 24 h. The age of adults at mating was either < 24 h or 10-day-old (delayed). No significant difference was observed between treatments. Box plots indicate the interquartile range (IQR; middle 50%), the median, and the data spread (1.5 times the IQR)

Unlimited mating significantly extended the pre-oviposition period (Wilcoxon rank-sum test: *W* = 48,* P* = 0.008), but there were no significant differences in the oviposition period (*W* = 68.5,* P* = 0.182), post-oviposition period (*W* = 126.5,* P* = 0.185) (Table 1), fecundity (*W* = 81,* P* = 0.460), offspring survival (Chi-square test: χ^2^ = 0, *df* = 1,* P* = 1), offspring sex ratio (χ^2^ = 0, *df* = 1,* P* = 1) (Table 2), or egg size (*W* = 638,* P* = 0.317) (Fig. 4) between females mated for 24 h and those with unlimited mating opportunity.

**Table 1:** Influence of mating opportunity and delayed mating on *Phytoseiulus persimilis* female reproductive periods (mean days ± SEM)

Mating opportunity	*n*	Pre-oviposition	Oviposition	Post-oviposition
Unlimited	15	1.9 ± 0.1**	18.2 ± 0.4	8.3 ± 1.8
Limited	13	1.4 ± 0.1**,*	17.0 ± 1.1	14.5 ± 3.5
Limited (delayed)^†^	15	1.8 ± 0.1*	17.1 ± 1.2	6.3 ± 2.6

Asterisks indicate significant differences as determined by the Wilcoxon rank-sum test. ** (*P* < 0.01) Indicates significant differences in the pre-oviposition period between individuals with unlimited and limited mating opportunities. * (*P* < 0.05) Indicates significant differences in the pre-oviposition period between individuals with limited mating opportunities, with and without a delay in the onset of mating. † Individuals were delayed for 10 days after reaching maturity before being allowed to mate

Individuals given limited mating opportunities were allowed to mate for 24 h. The age of adults at mating phase was either < 24 h or 10-day-old (delayed)

**Table 2:** Influence of mating opportunity and mating delay on fecundity (mean eggs ± SEM), offspring survival (%), and sex ratio (% of females) of *Phytoseiulus persimilis* females

Treatment	Fecundity	Survival (%)	Sex ratio (%)
Unlimited	73.9 ± 1.7	86.7 ± 6.2	84.0 ± 6.7
Limited	70.8 ± 3.9	90.0 ± 5.5	81.5 ± 7.1
Limited (delayed)^†^	67.5 ± 4.9	96.7 ± 3.3	82.8 ± 6.9

Individuals given limited mating opportunities were allowed to mate for 24 h. The age of adults at mating phase was either < 24 h or 10-day-old (delayed). The sample size (*n*) is 30 for both the sex ratio and survival per frequency. No significant difference was observed between treatments

Individuals (^†^) were delayed for 10 days after reaching maturity before being allowed to mate

### Experiment 1b: Influence of delayed mating on female reproductive traits and offspring survival

Delayed mating prolonged the pre-oviposition period of females with limited mating opportunity (Wilcoxon rank-sum test: *W* = 57,* P* = 0.029), but no significant differences were observed in oviposition period (*W* = 77,* P* = 0.350), post-oviposition period (*W* = 136.5,* P* = 0.074) (Table 1), fecundity (*W* = 92,* P* = 0.818), offspring survival (Chi-square test: χ^2^ < 0.001, *df* = 1,* P* = 1), offspring sex ratio (χ^2^ = 0.268, *df* = 1,* P* = 0.605) (Table 2), or egg size (*W* = 479,* P* = 0.236) (Fig. 4).

### Experiment 2: Influence of male copulation history on lifespan, female reproductive traits, and offspring survival

**Fig. 5:**
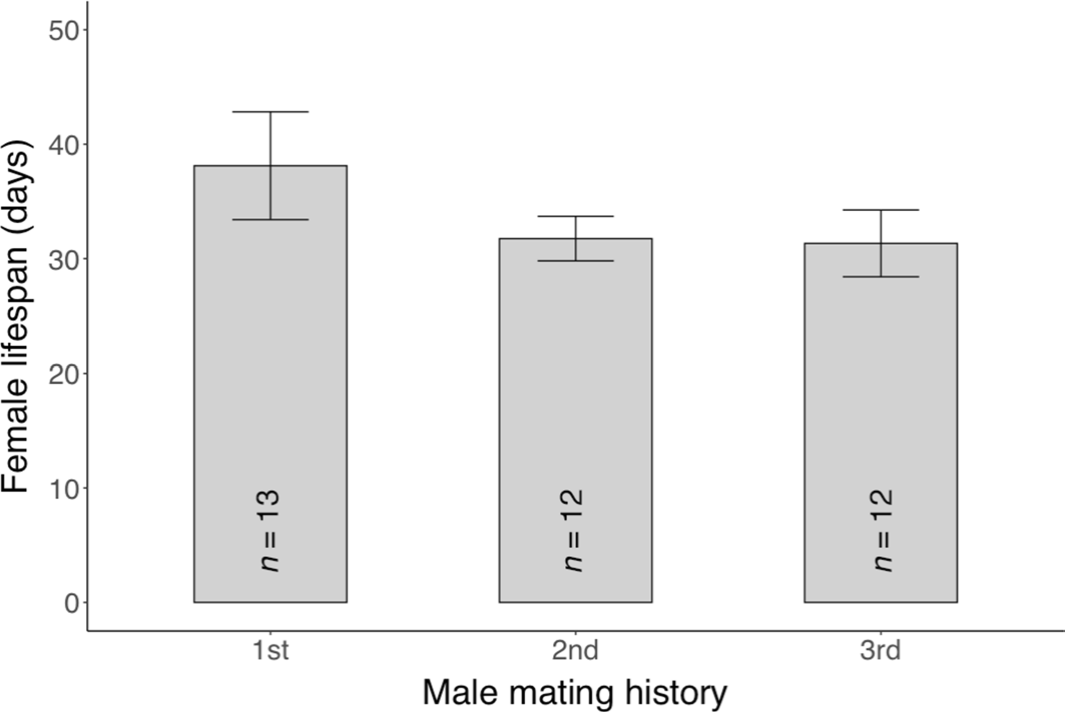
The lifespan of *Phytoseiulus persimilis* females mated with males with different mating histories (copulation frequency). No significant difference was observed between treatments. Box plots indicate the interquartile range (IQR; middle 50%), the median, and the data spread (1.5 times the IQR)

**Fig. 6:**
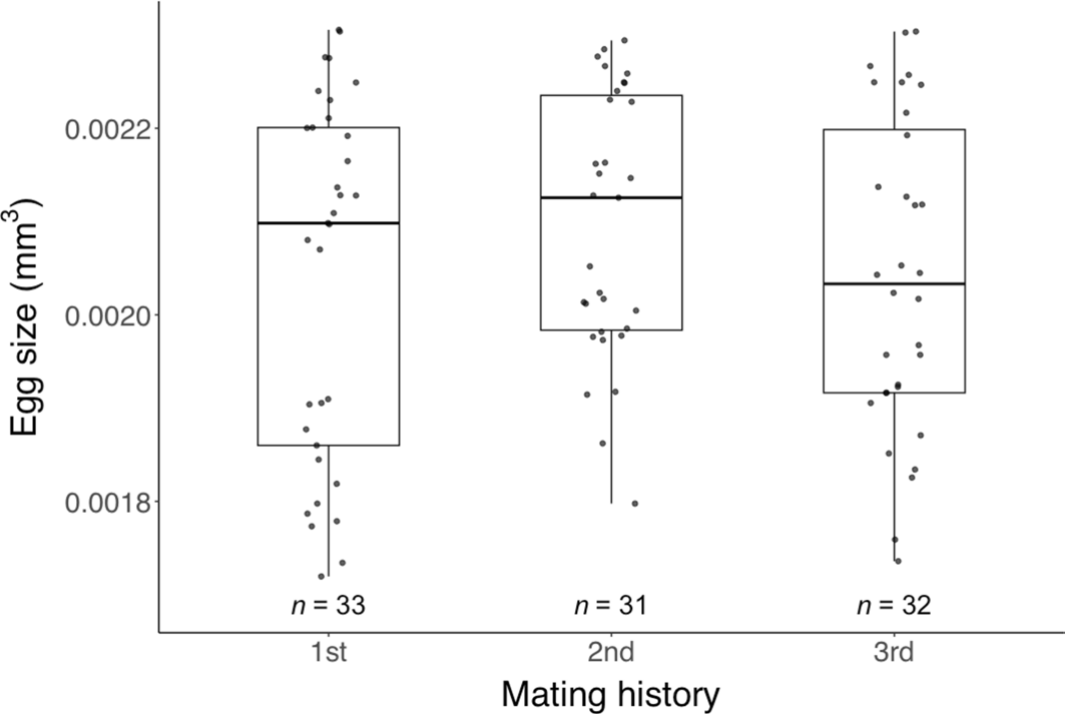
Influence of male copulating frequency on the egg size (individual volume) of *Phytoseiulus persimilis*. No significant difference was observed between treatments. Box plots indicate the interquartile range (IQR; middle 50%), the median, and the data spread (1.5 times the IQR)

Male copulation history did not significantly affect the lifespan of females (ART ANOVA: *F*_2,34_ = 1.106,* P* = 0.342) (Fig. 5) nor female reproductive traits, including pre-oviposition period (*F*_2,34_ = 0.833,* P* = 0.443), oviposition period (*F*_2,34_ = 0.424,* P* = 0.658), and fecundity (*F*_2,34_ = 0.192,* P* = 0.826) (Tables 3 & 4); and it also did not affect offspring traits, including sex ratio (Chi-square test: χ^2^ = 0.349, *df* = 2,* P* = 0.840), survival rate (χ^2^ = 1.071, *df* = 2,* P* = 0.585) (Table 4), and egg size (ART ANOVA: *F*_2,93_ = 0.996,* P* = 0.373) (Fig. 6).

**Table 3:** Influence of male copulation history on *Phytoseiulus persimilis* female reproductive periods (mean days ± SEM)

Frequency	*n*	Pre-oviposition	Oviposition	Post-oviposition
First mate	13	2.1 ± 0.2	17.2 ± 1.8	14.4 ± 4.7
Second mate	12	1.8 ± 0.1	17.9 ± 0.7	7.2 ± 2.2
Third mate	12	1.9 ± 0.4	17.2 ± 1.1	7.8 ± 2.7

No significant difference was observed between treatments

**Table 4:** Influence of male copulating history on fecundity (mean eggs ± SEM), offspring survival (%), and sex ratio (% of females) of *Phytoseiulus persimilis* females

Frequency	Fecundity	Sex ratio (%)	Survival (%)
First mate	67.8 ± 7.2	82.1 ± 7.0	93.3 ± 4.6
Second mate	71.8 ± 2.6	75.9 ± 7.8	96.7 ± 3.3
Third mate	67.7 ± 3.8	77.8 ± 7.6	90.0 ± 5.5

The sample size (*n*) is 30 for both the sex ratio and survival per frequency. No significant difference was observed between treatments

*Phytoseiulus persimilis* males mated 3 times over 3 days showed a statistically similar lifespan (39.1 ± 3.2 days) compared to those allowed to mate for 24 h (Wilcoxon rank-sum test: *W* = 123.5,* P* = 0.250). However, it was significantly longer than the lifespan of males given unlimited mating opportunity (*W* = 57.5,* P* = 0.040).

## Discussion

The current study investigated the influence of mating opportunities, mating delay, and male mating experience on lifespan (of both sexes), female reproductive traits, and offspring traits of *P. persimilis.* The findings indicate that variations in mating access and participation significantly affect the lifespan of *P. persimilis*. The results of this study align with previous studies showing that unmated *P. persimilis* females have almost double the lifespan of the mated ones (Amano and Chant 1977), a trend also observed in phytoseiid predators *N. californicus* (Gotoh and Tsuchiya 2008) and *Neoseiulus cucumeris* (Ji et al. 2007). Many animal species have observed a negative relationship between fecundity and lifespan (Adler et al. 2013). The energetic costs associated with reproduction may reduce the energy allocated to somatic maintenance and repair, diminishing survival and shortening lifespan (Nakagawa et al. 2012). However, in the current study, unmated males and males allowed to mate for 24 h exhibited similar lifespans. This may be explained by females’ investment in egg production after mating, whereas males would cease reproductive investment if additional mating was prevented, allocating remaining energy to somatic maintenance.

Our observations also revealed a sexually distinct response to increased mating access, contrary to initial predictions in our first hypothesis: females with unlimited mating opportunity showed similar lifespans to those with only 24-h mating access, whereas males with unlimited access to mates experienced significantly reduced lifespans. The non-significant effect on lifespan suggests that increased mating access in *P. persimilis* does not pose a substantial cost to females and may not act as a form of harassment, as observed in *T. urticae* (Li and Zhang 2021a, b). However, because we did not conduct continuous observation, it remains unclear whether repeated mating occurred in individuals with unlimited access. Females may have refused to mate, or males and females might have had minimal interactions during their lifetime (Schausberger et al. 2017). In contrast, the reduced lifespan in males with increased mating opportunities indicates a higher energetic cost of repeated mating, mating attempts, or other intraspecific interactions (Amano and Chant 1977, 1979; Xu et al. 2023). These costs may stem from behaviors such as mate-finding and sperm production (Amano and Chant 1977, 1979). In *N. cucumeris* males, multiple mating was also associated with a shorter lifespan compared to single mating (Ji et al. 2007).

Our results indicate that increased mating access does not enhance female fecundity in *P. persimilis*. This finding aligns with previous reports suggesting that repeated inseminations do not increase female fecundity in this species (Amano and Chant 1978; Schausberger et al. 2017). Contrary to our second hypothesis, increased mating access with the same male did not positively affect offspring traits, including egg size and survival. Further research exploring the effects of polyandry or multiple mates on offspring quality, noted in other species (Di et al. 2023), may provide greater insights into the reproductive biology of *P. persimilis*.

Delayed mating did not extend the lifespan of females, as reported for *T. urticae* (Li and Zhang 2021a). Instead, the influence of delayed mating on *P. persimilis* female lifespan was, as in our third hypothesis, similar to that seen in *N. californicus*, a phytoseiid predator with ecological parallels to *P. persimilis* (Gotoh and Tsuchiya 2008; Schausberger et al. 2017).

We found that the males of *P. persimilis* could inseminate at least three females across 3 days without a decline in female reproductive capability or impact on offspring traits. Given the female-biased sex ratio of *P. persimilis* (4:1) (Toyoshima and Amano 1998), mating with the first few females without a decrease in reproductive output would offer a reproductive advantage to males. However, male fertility may decline with age, as seen in various species (Johnson and Gemmell 2012; Vega‐Trejo et al. 2019), including *P. persimilis* (Amano and Chant 1978). Our findings differed from the predictions of our fourth hypothesis, and from the findings of Amano and Chant (1978), where females mated with already mated *P. persimilis* males had reduced fecundity compared to the initial female mate. However, this discrepancy may stem from differences in prey species (i.e., *Tetranychus pacificus* McGregor vs *T. urticae*) and pre-experiment rearing methodologies between studies. Furthermore, previous mating experience of male *N. cucumeris* significantly influenced the fecundity, oviposition period, and lifespan of females with varying trends (Ji et al. 2007). Whether males in species with a higher level of polyandry (e.g., *N. cucumeris*) exert a greater influence on female fecundity or exhibit more age and experience-dependent variation in sperm quality, compared to males in less polyandrous species (e.g., *P. persimilis*), requires further investigation.

Finally, and also in contrast to our fourth hypothesis, offspring sex ratios were found to be consistent across all treatments. Factors such as prey density (Toyoshima and Amano 1998), parental dietary condition (Han et al. 2024) and sperm quantity received (Lv et al. 2019) have been reported to influence sex ratios in *P. persimilis*. Thus, additional interactions with a single male (e.g., repeated mating) may not play a significant role in determining offspring sex ratios.

Although early offspring were randomly selected for survival assessment, egg size and survival could vary for later-produced offspring (Zhang et al. 2024). Since all offspring had access to abundant food in this study, future studies might consider exploring offspring performance (i.e., survival) under more stringent environmental conditions or when provided with a mixed diet that includes spider mites at various life stages (Fischer et al. 2011; Fox and Mousseau 1996). The size of *P. persimilis* females and males can influence mate choice and reproductive success, highlighting the importance of incorporating this factor into future investigations (Enigl and Schausberger 2004; Walzer and Schausberger 2014, 2015). Finally, we did not quantify the exact number of successful mating events or mating duration, factors known to influence fecundity in *P. persimilis* females (Lv et al. 2019; Schulten et al. 1978). Future studies with more precise mating event data could yield clearer insights into these reproductive dynamics. Overall, our results suggest that repeated mating provides no reproductive advantage but also imposes no observable costs on *P. persimilis* females.

## Data Availability

The original data that support the results of this research are available for open access from Manaaki Whenua—Landcare Research DataStore 10.7931/pmb2-hk03.
